# Microbiological Risk Assessment in Foods: Background and Tools, with a Focus on Risk Ranger

**DOI:** 10.3390/foods12071483

**Published:** 2023-03-31

**Authors:** Antonio Bevilacqua, Alessandro De Santis, Gaetano Sollazzo, Barbara Speranza, Angela Racioppo, Milena Sinigaglia, Maria Rosaria Corbo

**Affiliations:** Department of the Science of Agriculture, Food, Natural Resources and Environment (DAFNE), University of Foggia, Via Napoli 25, 71122 Foggia, Italy; alessandro.desantis@unifg.it (A.D.S.); gaetano_sollazzo.546187@unifg.it (G.S.); barbara.speranza@unifg.it (B.S.); angela.racioppo@unifg.it (A.R.); milena.sinigaglia@unifg.it (M.S.); mariarosaria.corbo@unifg.it (M.R.C.)

**Keywords:** risk, hazard, analysis, ranking, foodborne disease, food safety and quality

## Abstract

Risk assessment is an important phase of the food production path; it is strictly related to the processing chain as a necessary step for safe foods. This paper represents a contribution to understanding what is and how risk assessment could be conducted; it aims to provide some information on the structure of risk assessment, the tools for its identification and measurement and the importance of risk assessment for correct communication. In this context, after a focus on the background and on some commonly used tools (Risk Ranger, FDA-iRisk, decision tree, among others), the paper describes how to perform risk assessment through three case studies: lettuce (for *Listeria monocytogenes*), chicken salad (for *Escherichia coli*), and fresh egg pasta (for *Staphylococcus aureus*) in the first step, and then a comparison of risk for chicken salad contaminated by different pathogens (*E. coli* O157:H7, *Campylobacter* spp. and *Salmonella* sp.). As a final step, a critical evaluation of Risk Ranger was carried out, pointing out its pros and cons.

## 1. Introduction

Food safety is one the major goals to achieve worldwide; in fact, one of the most underestimated problems is the high incidence of foodborne diseases [1,2]. According to the World Health Organization (WHO), unsafe food products cause 600 million cases of foodborne diseases and 420,000 deaths each year, corresponding to the loss of 33 million healthy life years (DALYs); this problem is particularly relevant as ca. 30% of foodborne deaths occur among children under 5 years of age, and this number is likely an underestimation [3]. Therefore, it is necessary that all stakeholders in the food chain understand the importance of their work, the weight of their decision on consumers’ health, and their role in food security and safety [4].

In this context, academia should develop, design, and apply an effective safety management system based on correct risk analysis in order to prevent and reduce health and safety challenges and threats; however, food safety management requires a correct definition and understanding of basic requisites, starting from a correct use of the terms “hazard” and “risk”. These were defined for the first time during the joint FAO/WHO expert consultation (Geneva from 13 to 17 March 1995) as follows [5]:

- Hazard is a biological, chemical, or physical agent in, or condition of, food, with the potential to cause an adverse health effect.
- Risk is a probabilistic function of an adverse health effect and the severity of that effect.

According to these definitions, hazard is a qualitative concept while risk is a probabilistic concept because it is a statistical and mathematical estimate of the probability that a hazard can cause harm to the consumer’s health [6]. In this context, after the definition of hazards, experts should correctly estimate risks as a preliminary step to set possible corrective measures or preventive strategies to assure food safety and quality. HACCP (Hazard Analysis Critical Control Point), introduced in 1993 [7], was the first structured approach for the correct identification of possible hazards throughout the flow of food products which is achieved through the definition of critical control points (CCP) [8]; in addition, it includes a set of preventive measures in terms of variables and parameters to monitor to reduce hazard occurrence [9], or the use of controlling measures when hazards have occurred to keep these at an acceptable level or to avoid injuries to health [6]. Nowadays, HACCP is still a milestone in the food industry; however, it is linked to the qualitative concept of hazard and focuses on specific situations rather than considering the issue of hazard control and risk reduction at the global level. This issue could be addressed by the risk analysis methodology.

## 2. Risk Analysis in the Food Industry

Risk analysis is a fundamental step to reduce to an acceptable level the problems occurring during the food production process and which can cause problems for the consumers’ health; according to FAO/WHO [4], it consists of three components: risk assessment, risk management, and risk communication. These phases were defined as follows:

- Risk assessment is the scientific evaluation of potential, or known, adverse health effects resulting from human exposure to foodborne hazards.
- Risk management is the operative consequence of risk assessment, and it consists in understanding policies and selecting the appropriate actions to achieve ALOP (appropriate level of protection).
- Risk communication is an exchange of information, opinions, and data among all the stakeholders involved in risk analysis.

Risk assessment is a qualitative and quantitative process, and it can be performed according to different standards, such as the IEC 31010:2019 or FAO/WHO guidelines [10]. It is composed of three main steps: risk identification, risk analysis, and risk evaluation.

Identification starts from a study about the target food and is usually based on scientific data, expert opinions, and previous experience [11]. Thanks to an accurate knowledge of the matrix, it is possible to identify biological, chemical, and physical hazards; some examples are reported in Table 1.

Each risk is analysed to understand its nature, severity, and consequences, focusing on many factors and variables (uncertainties, likelihood, consuming scenario etc.). One of the most used techniques is the magnitude/likelihood matrix, which allows numerical values to be set for the magnitude and likelihood of the risk [12] and to display a rank for that risk in an intuitive graphical way; magnitude (M) is a measure of the harmful consequences of the risk, while likelihood (L) refers to the chance of something happening; thus, it is usually described using terms such as probability or frequency. According to this approach, risk level is the result of the following formula:

Risk Level = Magnitude × Likelihood

The magnitude/likelihood matrix is based on consequences scales which associate the magnitude and the likelihood of the risk to a numerical value (Table 2). After giving a value to these two factors, the results give a risk estimation (or risk level), as shown in Table 3; colour defines the risk weight as follows:

- Green cells represent a low risk level.
- Yellow cells represent a middle risk level.
- Red cells represent a high risk level.

According to this matrix, authorities and participants of the food chain will focus on the higher risk level to find the right preventive strategies and to reduce the risk to an acceptable level.

In the food industry, the biological and chemical risks are the most feared because of the damage which they can cause to human health; the following sections of this paper address the topic of microbiological risk. For this purpose, the Codex Alimentarius Commission published a set of guidelines (CXG 30-1999) [14], while the FAO (Food and Agriculture Organization) and WHO (World Health Organization) released “Microbiological risk assessment: guidance for food” (MRA 36) [10], which updates and brings together in a single volume three previous guidance documents (MRA 3, MRA 7, and MRA 17) (MRA; Microbiological Risk Assessment) [15].

## 3. Risk Assessment for Microbiological Issues in the Food Industry

**Figure 1:**
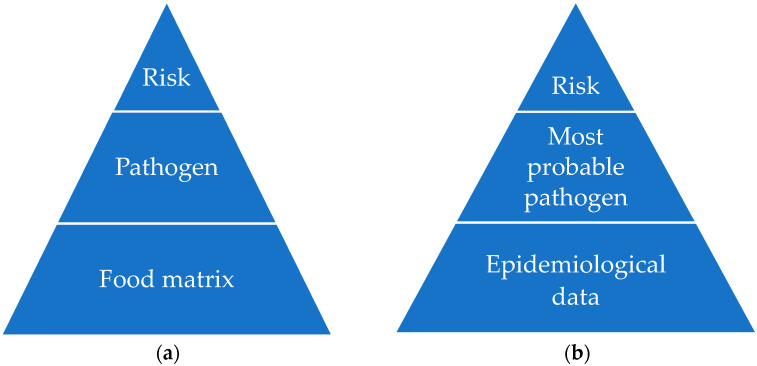
Graphical representation of the top-down (**a**), and bottom-up (**b**) approaches.

The MRA is a specific set of guidelines to manage microbiological risk in the food industry. Two different approaches are possible: bottom-up or top-down (Figure 1). In the top-down approach, the study is based on the knowledge about the main hazard which can occur in a food matrix, while the bottom-up approach uses epidemiological information to identify the most probable hazards and to assess the risk [16].

**Figure 2:**
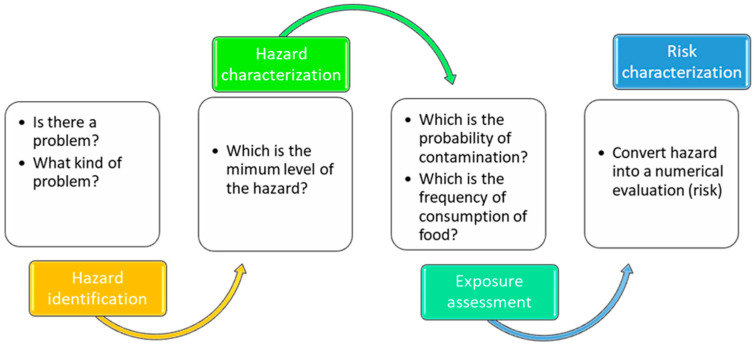
Steps and questions in the Risk Assessment process.

The MRA is divided into four phases, and each phase can be summarized by a set of a few questions. In the following paragraphs, all MRA steps [15] are briefly summarized, as shown in Figure 2.

### 3.1. Hazard Identification

The aim of this first phase is to identify all the pathogens which can survive in the target food and which can be dangerous for consumers’ health; it is worth mentioning that the more correct the microorganism identification is, the more effective is the risk assessment. In this step, assessors should consider the properties of the food matrix (composition, intrinsic, and extrinsic factors), its technological story (possible thermal treatments or any other process which could affect pathogen survival), and pathogen physiology, as well as the main properties of the raw material and its origin.

Hazard identification, like any other stage, can be characterized by data and methodologies validated at the international level (for example, the surveys periodically performed by regulatory agencies; the tools of Predictive Microbiology, such as ComBase; or CB Premium) and supported by the experience of risk assessors or by the latest advances in the literature.

### 3.2. Hazard Characterization

The second phase consists of a qualitative and quantitative evaluation of the severity degree of the pathogen, considering the nature of issues such as fever, diarrhoea, and neural problems, among others. In this context, details of the severity of pathogens can be found in some reports of public agencies on foodborne diseases periodically published at the international level; an example is the report on zoonosis by the European Union [17]. The details required for this step are, among others, the kind of disease (infection or toxin production), the infectious dose, the pathways of disease (how the pathogen enters the host and the mechanisms involved in disease progression), host susceptibility, treatments required for disease remission, and epidemiological data (hospitalization, medical treatments, deaths).

### 3.3. Exposure Assessment

The third phase supplies information about the level of danger posed by the pathogen or toxin present in food during consumption, considering the different potential paths or moments of contamination, the impact of the various processing steps, the kind of food (raw material or processed food), and any relevant data related to the frequency and the quantity of consumption. In this step, assessors should focus on the flowchart of food production and point out all the hurdles that a pathogen could encounter (e.g., thermal treatments, acidification, storage, preparation before consumption); it is important to precisely define all conditions and their quantitative effect on the pathogen. It is also important to have access to survey data on the contamination of the raw material.

### 3.4. Risk Characterization

In this phase, the previous phases are integrated to gain a qualitative and/or quantitative evaluation of the risk as a background for correct risk management decisions. Generally, all details and information found throughout the hazard identification, hazard characterization, and exposure assessment steps are used as input values for algorithms or other modelling tools.

Risk assessment can be qualitative, semi-quantitative, and quantitative; qualitative assessment compensates for the lack of data by using expert opinions and intuitions, while quantitative risk assessment can be stochastic or deterministic; it is only based on strong and large data and/or statistics such as epidemiological reports, consumption rate, burden of disease, etc. and needs the support of specific software to collect, analyse and interpret the large amount of data.

The semi-quantitative risk assessment is a combination of qualitative and quantitative assessments, as each qualitative risk estimate is assigned a number which has a statistical weight, such as for probability ranges. One of the most common ways to represent semi-quantitative assessment is the risk matrix.

## 4. Risk Ranking Tools

There are many tools designed for risk ranking; in 2015, the Panel on Biological hazards (BIOHAZ) of EFSA summarised the performances of eight risk ranking tools [18]:

1. Decision tree;
2. Pathogen–produce pair attribution risk ranking tool (P^3^ARRT);
3. Food of non-animal origin risk ranking tool (EFoNAO-RRT);
4. Risk Ranger;
5. MicroHibro;
6. Swift quantitative microbiological risk assessment (sQMRA);
7. FDA-iRISK;
8. Communicable Diseases in Europe (BCoDE) toolkit.

Each tool has its pros and cons, as reported in Table 4.

This paper briefly describes three tools based on, and representative of, three different approaches: qualitative (Decision tree), semi-quantitative (Risk Ranger), and quantitative (FDA-iRisk) tools.

### 4.1. Decision Tree

This tool is usually based on a flowchart with the possibility of choices being yes or no for each question. It is a full qualitative tool [19], and its main benefit is the possibility to adapt it according to the type of product, context, and needs of the food company. Thanks to its flexibility, the decision tree is used as the common basis for many different algorithms such as C4.5, CART, and SPRINT [20], thus opening new ways for using it to lead risk analysis in a wide variety of scientific fields, such as medical [21,22], financial [23], and food-related industries [24].

**Figure 3:**
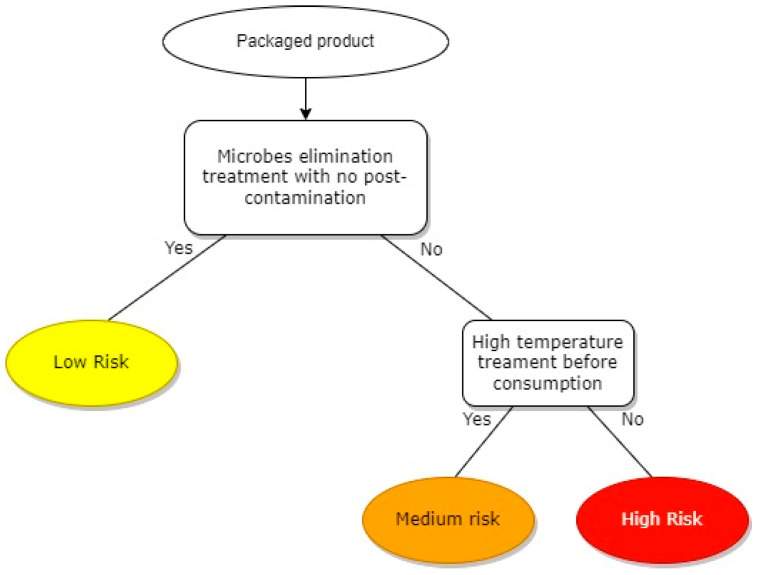
Example of a decision tree.

Figure 3 shows an example useful to most food industries. The final output of the tool allows the classification of risks into three different levels: low (yellow), medium (orange), or high (red).

Because of its qualitative nature, another benefit is its efficiency, even though quantitative data are missing. Furthermore, the diagram shape contributes to sharing data and ideas to all stakeholders involved in the risk analysis in a simple and intuitive way. On the other hand, the main limit is the high level of subjectivity as experts can have different opinions or knowledge about the matter, and this can give uncertain and/or incorrect outputs.

### 4.2. Risk Ranger

Risk Ranger is a semi-quantitative risk assessment tool developed by the University of Tasmania, released in 2002 as a spreadsheet [25]; nowadays, it is available on the website CBpremium.com [26].

In Risk Ranger, assessors have to answer eleven questions of a qualitative and quantitative nature about food safety, and the tool releases four outputs, as follows:

1. Probability of illness per consumer per day.
2. Total predicted illness/year in the population of interest.
3. Comparative risk in the population of interest.
4. Risk ranking.

The last parameter is the most user-friendly among the four outputs because it is a coloured output, easily understandable by all stakeholders. A comprehensive description of this tool is found in Section 5.

### 4.3. FDA-iRisk

FDA-iRisk is a quantitative risk assessment tool developed by the US Food and Drug Administration (FDA) with the support of American and foreign group of experts, and it is useful for estimating microbial and chemical risks [27]. It is a website-based software [28] which needs different data related to hazard severity, food production, food assumption, and dose-response effect, among others. It is based on process models (initial contamination, production/processing/handling steps), logical connections, dose-response relationships, probability density, growth or inactivation models for microorganisms and Monte Carlo simulations. One of the most important tools is the Disability-Adjusted Life Year (DALY), which indicates the time lived in a disabled condition or the time lost because of an early death caused by the assessed risks [29]. This index is one of the most important parameters to identify the biological hazards which cause the highest risk to consumers’ health [30].

FDA-iRISK supports the following risk (exposure) scenarios:

- Acute exposure to microbial hazards in a single food.
- Acute exposure to chemical hazards in a single food.
- Chronic exposure to chemical hazards in a single food.
- Chronic exposure to chemical hazards in multiple foods (multifood).

**Figure 4:**
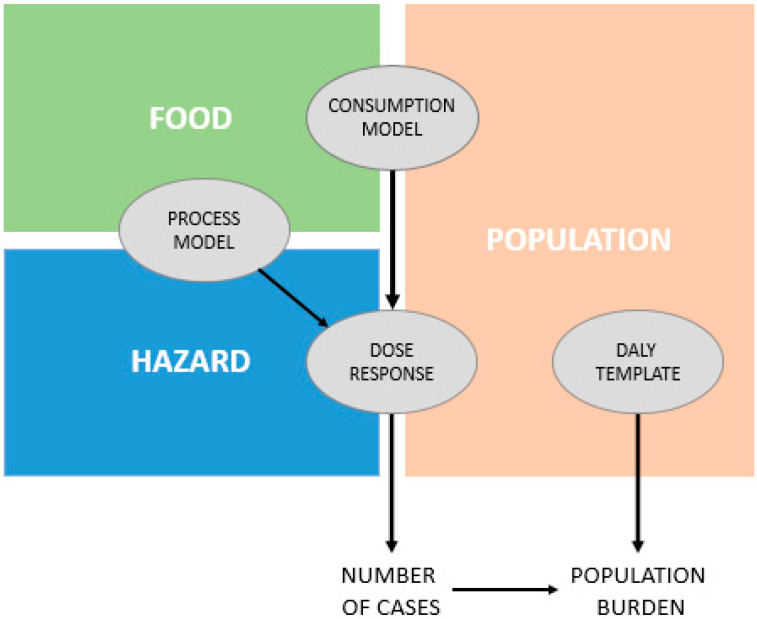
The seven elements of FDA-iRisk and their relationships in a generic risk scenario (modified from [31]).

Risk assessors choose the type of risk scenario and set it by addressing seven elements, which are completely editable according to necessity and the available data (Figure 4); the seven elements are:

- Food.
- Hazard.
- Population of consumers.
- Process model (i.e., food production, processing and handling practices).
- Consumption pattern(s) in the population.
- Dose-response relationship(s).
- Burden of disease measures associated with different adverse health effects from the hazard (i.e., a health metric such as losses in DALYs).

The last four elements must be set using numeric parameters and are data mined by risk assessors. Moreover, it is worth mentioning that some of these parameters only need single values while others offer the possibility of specifying uncertainty by using variability distribution [27].

FDA-iRisk generates two types of outputs:

- Risk Estimates and Scenario Ranking: this creates a report with risk estimates and ranking results for one or more scenarios, including full documentation of model inputs.
- Summary of Model Elements: this creates a report summarizing model elements with no risk estimates. The scenarios are not computed.

**Figure 5:**
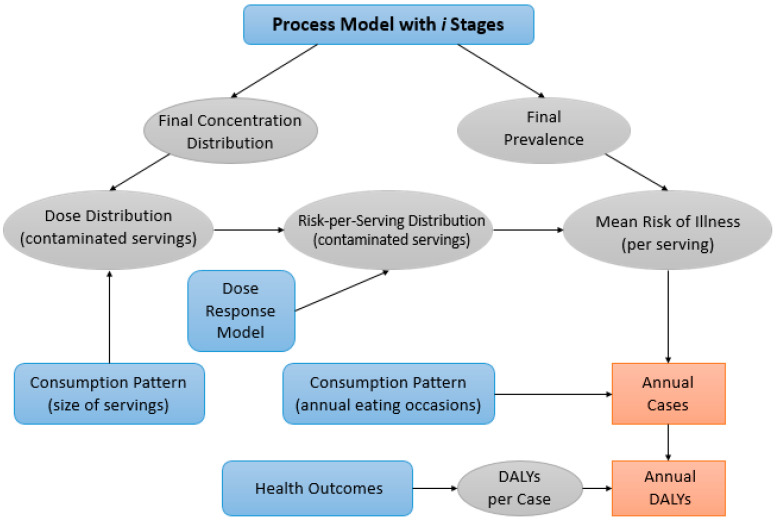
Illustration of inputs and outputs for a microbiological risk scenario in a food (modified from [31].

Figure 5 is a graphical summary of necessary inputs (square nodes) and expected outputs (circle nodes) for a microbial risk scenario.

The main benefit of this tool is the wide variety of foods, hazards, and risks which can be analysed and assessed [32], along with the correct definition of the scenario. On the other hand, the necessity for a large amount of data [27] is also, according to the authors of the present review, a weakness of the tool. These data can be difficult to find or not be available for a food company; moreover, the usability of the tool is quite difficult for non-expert assessors. Even though the outputs gained through this tool are significant, the resources required to train risk assessors and the time necessary to find data cannot be supplied by many food companies.

## 5. Risk Ranger in the Food Company Context

As reported previously, various methods and tools can be used for microbiological risk assessment. Focusing on the strength and weakness factors described in Section 4, according to the authors’ experience, the most suitable solution for a food company appears to be Risk Ranger because it combines a simple way to display all elements of the tool and the mathematical and statistical basis behind it. Furthermore, the model can be used by risk managers to think in terms of risk and to simulate the effect of different risk reduction strategies.

To better explain the usability of this tool and its feasibility, some case studies were performed.

Once logged in, the tool guides users through 11 questions grouped into three sections:

- Susceptibility of the population and severity of the pathogen.
- Probability of consumption of the contaminated food by the consumer (exposure).
- Probability that the contaminated food contains an infectious dose.

The answers are usually qualitative because they allow the user to choose between different hypotheses, except in some cases; the answers are converted by the software into numerical values and become inputs for mathematical formulas. The result, called risk ranking, is a number between 0 (no risk) and 100 (maximum risk of consumption of the total population of interest of the food product contaminated by a lethal dose of the pathogen) and expresses the likelihood and the severity of product–pathogen–processing combinations, while other outputs are the probability of illness per day per consumer of interest, the total predicted illnesses per annum in the population of interest, and the comparative risk in the population of interest.

The eleven questions relate to the following [26]:

1. Hazard severity (severe, moderate, mild, or minor hazard, depending on the need for medical intervention and/or patients’ death).
2. How susceptible the population of interest is, to better define the target of the pathogens (from general population to some groups).
3. Frequency of consumption (daily, weekly, monthly, a few times per year or other measures given by user).
4. Proportion of the consuming population (from a low percentage of the target population to 100%).
5. Size of the consuming population, where the user can add the size of the population of interest.
6. Probability of contamination of raw product per serving (from less than 0.01% to the worst scenario approach, where the raw material is always contaminated; that is, 100%).
7. Effects of food processing, with the possibility of a focus on the flowchart and on the existence of some steps able to significantly reduce or increase levels of the pathogen.
8. Potential post-processing recontamination (yes or no, depending on the flowchart).
9. Importance of control processes after food processing (from “well controlled” to “gross abuse occurs”, depending on how the product is stored before preparation and consumption).
10. Level of increase in post-processing contamination increase level (the increase in the pathogen level during post-processing which can cause negative effects to average consumers).
11. Effect of preparation before eating (if a kind of preparation is required before consumption).

### 5.1. Microbiological Risk Assessment: Practical Cases

Three different foods were studied as follows:

1. *Listeria monocytogenes* in ready-to-use lettuce [33];
2. *Escherichia coli* in chicken salad [34];
3. *Staphylococcus aureus* in fresh egg pasta [35].

Table 5 shows the answers set by assessors, and the outputs of the tool; generally, the choices for the different questions were based on worldwide habits while the target was set to the Italian population to gain relevant results. The hazard was set to minor for *Staph. aureus*, due to the low grade of hospitalization and disease severity, and mild for *E. coli*, as the focus was on the overall strains and not only on the O157:H7 serotype. On the other hand, for *L. monocytogenes,* the choice was “moderate”, as the targets mostly exposed are pregnant women/foetuses and aged people. Based on the average habits for Western countries, the frequency of consumption was set to “weekly” for fresh egg pasta and chicken salad, and twice a week for lettuce. For the contamination of raw material, the option “sometimes” or “infrequent” was set, depending on the authors’ knowledge of the epidemiology of the three pathogens, while the other inputs take into account that pasta and chicken are usually cooked, while lettuce is not. The final ranking was 59 for *L. monocytogenes* in lettuce, which means a risk level requiring controlling measures; this rank probably depends on certain inputs (frequency of consumption, proportion of consuming population, post-processing, and possibility of recontamination).

For the *E. coli* in chicken salad and *Staph. aureus* in fresh pasta, the risk ranking was 40, which means a lower risk level requiring some preventive or controlling measures. The tool offers other outputs, namely, the probability of illness per consumer per day, the total predicted illness per annum in the population of interest, and the comparative risk in the population of interest. The probability of illness per consumer per day is not strictly a measure of risk, because it does not consider the severity of disease, and it is only based on the “probability of a disease-causing dose being present in a portion of the product of interest” and on the exposure; it is in the range of 0–1 and measures the probability of a customer being affected by the disease. In the conditions used in this paper, the value was the highest for the combination of *L. monocytogenes*/lettuce (2.50 × 10^−6^) and the lowest for *Staph. aureus*/pasta (4.27 × 10^−7^).

The second output is the “total predicted illness per annum in the population of interest” (from 7.02 × 10^3^ in pasta/*Staph. aureus* to 1.17 × 10^5^ for *L. monocytogenes*/lettuce); this index is probably the most understandable measure as it offers a prediction of the possible cases of illness due to that food. For the conditions presented in this paper, the output was probably overestimated due to some input conditions and to the use of a worst scenario approach. Finally, the “comparative risk” is a measure of relative risk, independent of the size of the population, but it relies on the size of the consuming population (75%). This last output is probably the most useful factor for measuring the risk for different combinations of pathogen/food, as well as for different populations.

Table 5 shows some examples of the use of Risk Ranger and how the different inputs could strongly affect outputs. It is worth mentioning that the correct use of this tool should be based on a higher number of food/pathogen combinations, and different pathogens should be evaluated for each food to assess the effective risk ranking and the pathogen requiring urgent controlling or preventive measures.

As an example, for chicken salad, the simulation was also performed for *Campylobacter* spp. [36,37], *E. coli* O157:H7 [38], and *Salmonella* sp. [39]; for the first two pathogens, the severity was set to moderate, while for *Salmonella* sp., the choice was set to “mild”. The risk ranking was 52 for *Campylobacter* spp. and *E. coli* O157:H7 and 40 for *Salmonella* sp., with a comparative risk from 3.21 × 10^−9^ to 3.21 × 10^−11^, suggesting that, at least for the pathogens hereby reported, *Campylobacter* spp. and *E. coli* O157:H7 are limiting for chicken salad safety.

### 5.2. Perspective and Limitations of Risk Ranger

Compared to decision trees and FDA-iRisk, Risk Ranger is a more suitable tool for a food company because of its ready-to-use characteristics and the statistical values of the outputs.

Risk Ranger is also a communication tool because the outputs work like a traffic light (green, yellow, red). However, as with all other tools, it has pros and cons, which are summarized in Table 6. The most important benefits include the user-friendly interface, the possibility of using it for a wide variety of foods, the combination of both qualitative and quantitative inputs, and the quantitative outputs, which could be used to implement preventive and/or corrective measures by assessors and public agencies. On the other hand, as with all tools, Risk Ranger has some limitations, including the fact that uncertainty is not assessed, and that there are two scenarios which are nonsense situations for risk assessment; namely, zero risk, which does not exist, and risk at 100%, which gives certainty and not probability. Furthermore, several situations could not be properly described by the eleven questions, as the ranking among the possible choices is too vague or does not allow the setting of intermediate values (for example, the proportion of the consuming population cannot be set between 25% and 75%).

Nevertheless, Risk Ranger has some potentialities with interesting perspectives in the future for education or training in academia. From a practical point of view, the exploitation of this tool at two levels (non-expert or academic users, and expert users) could be interesting. For training or non-expert users, the tool can be used in the current version with a few adjustments (for example, by implementing choices in some questions) or by adding the possibility of choosing among pathogens in the first question, thus reducing the misunderstanding of risk severity by non-expert users.

For the second level (expert interface), the tool could be connected to other tools/databases of Predictive Microbiology; for example, when users are requested to choose if (and to what extent) the process does or does not eliminate the pathogen. This connection could reduce the subjectivity in addressing the different options, thus adding an input derived by a mathematical simulation.

Despite these limitations, the authors are of the opinion that Risk Ranger could have some important perspectives connected to its use as a training tool and a risk tool for food companies to understand the effect of each change in the food chain on food safety, as well as for regulatory agencies to set guidelines and recommendations.

## 6. Conclusions

Risk assessment represents a fundamental process in the estimation of the microbiological risk associated with food ingestion and is an important field of research, both for policy makers and food companies. However, it is still perceived by some stakeholders, mainly by food companies, as a complex procedure suitable only for academia or public agencies, with few practical implications.

This paper addresses some issues and key points in the definition of risk and its origin, as well as how to use simple tools, such as Risk Ranger, to allow for an effective assessment even by non-expert users; in addition, the issues are considered from the point of view of food companies and on the practical implications of risk assessment. The description of Risk Ranger and the case studies of three products show that a food company can set up a more structured and accurate risk management system to address policy makers’ and consumers’ requests in terms of food safety; at the same time, a good risk assessment allows less expensive risk-reduction strategies to be found. Moreover, this paper also offers an overview of the pros and cons of Risk Ranger and future perspectives to improve its functionalities for companies or for training purposes.

In conclusion, risk assessment is still very difficult to understand for many food producers, consumers, and stakeholders in general, but this paper could contribute to the diffusion of a new awareness toward this topic.

## Figures and Tables

**Table 1:** Examples of biological, chemical, and physical hazards in the food industry.

Biological Hazard	Chemical Hazard	Physical Hazard
Bacteria	Pesticides	Foreign bodies
Viruses	Heavy metals	Insects
Protozoa	Toxins	Employees’ personal items

**Table 2:** Magnitude and likelihood scales [13].

Rating	Magnitude	Likelihood
5	Lethal	Expected likely
4	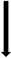	** 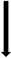 **
3		
2		
1	Irrelevant illness	Extremely unlikely

**Table 3:** Magnitude/likelihood matrix and risk level. Red, high risk; yellow, medium risk; green, low risk.

Magnitude rating	5	5	10	15	20	25
	4	4	8	12	16	20
	3	3	6	9	12	15
	2	2	4	6	8	10
	1	1	2	3	4	5
		1	2	3	4	5
		Likelihood rating				

**Table 4:** Resumé of advantages and limitations of evaluated tools by BIOHAZ in 2015 [18].

Tool	Pros	Cons
Decision tree	- Simple data management - Useful with poor quantitative data - Great adaptability for all users	- Imprecision - Some factors cannot be evaluated - Incomparability of outputs with semi-quantitative and quantitative risk tools
P^3^ARRT	- User-friendly interface - Minimal training needed - Periodic update of databases	- Necessity for frequent updates - Lack of data is handled by augmenting risk evaluation
EFoNAO	- Easy communication of multi-criterion model - Possible use of qualitative and uncertain input	- Some factors cannot be used as input data - Much effort required for manual handling to insert data
Risk Ranger	- Meaningful output - Simple and easy to use - Useful for a wide variety of food matrices and pathogens - Clear communication of outputs - Possible conversion to a probabilistic model	- Uncertainty and variability not evaluated - Some information cannot be entered
MicroHibro	- Advanced user interface - Predictive growth model included in the tool - Designing possibility of all production phases - Web-sharing of results	- Limited number of outputs - Slow Monte Carlo’s calculation process
sQMRA	- Both probabilistic and stochastic outputs - Predictive growth model included in the tool	- Limited number of probability distributions - Complex output file management
FDA-iRisk	- Advanced interface - Wide variety of inputs - Possibility to choose between deterministic or stochastic analysis - Combination of different scenarios and hazards	- Overestimation of risk - Deep knowledge of risk assessment inputs required
BCoDE	- Meaningful outputs - Reduced complexity due to limited number of inputs - Advanced user-friendly interface	- Unable to take into account pathogen transmission pathways

**Table 5:** Answers to the questions in Risk Ranger. Answers given following authors’ knowledge and information found in references [33,34,35]. Simulation was performed for a general audience; the exception was for question 5 (size of consuming population), where the input was based on the Italian population to gain realistic outputs.

*L. monocytogenes* in Ready-to-Use Lettuce	*E. coli* in Chicken Salad	*Staph. aureus* in Fresh Egg Pasta
Susceptibility and Severity		
*1. Hazard severity*		
**Moderate hazard**—The risk is medium because *Listeriosis requires* hospitalisation in most cases.	**Minor hazard**—The patient rarely requires medical assistance	**Mild hazard**—The patient rarely requires medical assistance
*2. Susceptibility of the population of interest*		
**Slight or very**—The subjects mostly affected are pregnant women/foetuses and aged people.	**General**—The pathogen can affect in a similar way all members of the population.	**General**—The pathogen can affect in a similar way all members of the population.
**Probability of exposure to food**		
*3. Frequency of Consumption*		
**Other (100 days per year, i.e., twice a week).**	**Weekly**—Consumption is generally once a week (average of consumers’ habits worldwide).	**Weekly**—Fresh egg pasta is not consumed daily.
*4. Proportion of consuming population*		
**Most**—Lettuce is eaten by most of the population	**Most**—Chicken salad is eaten by most of the population	**Most**—Fresh egg pasta is eaten by most of the population, at least for countries where pasta is generally consumed
*5. Size of Consuming Population*		
**60,000,000**: the test was carried out taking into consideration the population of Italy		
**Probability of food containing an infectious dose**		
*6. Probability of Contamination of Raw Product for Serving*		
**Infrequent**—The lettuce is not necessarily contaminated if correct agronomic practices have been carried out or certified and controlled water has been used.	**Infrequent**—The probability of contamination inside raw chicken meat is low.	**Sometimes**—The main source of contamination is human handling and contaminated food contact surfaces
*7. Effect of Processing*		
**The process slightly reduces hazards**—Lettuce is processed using chlorine.	**The process usually eliminates hazards**—Meat processing (blanching and/or cooking) reduces the presence of pathogens.	**The process usually eliminates hazards**—All the process phases (for example pasteurization), if correctly carried out, reduce the presence of pathogens (at least, of viable cells).
*8. Potential for recontamination after processing*		
**None**—Generally the probability is very low.	**None**	**Yes, minor**—Generally the probability is very low; however, a minor risk was assumed based on the worst scenario approach (contaminated surfaces)
*9. Effectiveness of the post-processing control system*		
**Not controlled**—*L. monocytogenes* is a psychrotrophic microorganism; thus, refrigeration cannot control it.	**Controlled**—Refrigerated storage usually delays pathogen growth.	**Controlled**—Refrigerated storage usually delays pathogen growth.
*10. Increase in levels of post-processing contamination*		
**None**	**None**	**None**
*11. Effect of preparation before eating*		
**No effect**—Lettuce does not require any processing before being consumed.	**No effect**—Since the chicken is pre-cooked, it would not require preparation before eating.	**Usually eliminates**—The boiling before consumption eliminates all bacteria; if a simulation on toxin is performed, it is worth mentioning that toxins are thermostable and cannot be eliminated during cooking.
*Risk Ranking*		
**59**	**40**	**40**
*Probability of illness per consumer per day*		
**2.50 × 10^−6^**	**4.27 × 10^−7^**	**4.27 × 10^−7^**
*Total predicted illness per annum in the population of interest*		
**1.17 × 10^5^**	**7.02 × 10^3^**	**7.02 × 10^3^**
*Comparative risk in the population of interest*		
**5.34 × 10^−8^**	**3.21 × 10^−11^**	**3.21 × 10^−11^**

**Table 6:** Pros and cons of Risk Ranger.

Pros	Cons
Suitable for expert and non-expert risk assessors	Less precise than quantitative tools
Simple user interface	Presence of two incorrect output values (Risk = 0%, as a zero-risk scenario, does not exist; and Risk = 100%, which represents a certainty and not a probability)
Ready-to-use	Uncertainty cannot be evaluated
Different food matrix can be analysed	Questions cannot include all possible situations and some choices are not possible
Suitable for different types of food companies	Tool not connected to other databases or models of Predictive Microbiology
Contemporary use of qualitative and quantitative data	
Statistical value of results	
Clear meaning of outputs	
Communications capability	
Better description of risk than qualitative tools	

## Data Availability

Not applicable.
